# Cyclotron production of ^225^Ac from an electroplated ^226^Ra target

**DOI:** 10.1007/s00259-021-05460-7

**Published:** 2021-07-01

**Authors:** Kotaro Nagatsu, Hisashi Suzuki, Masami Fukada, Taku Ito, Jun Ichinose, Yoshio Honda, Katsuyuki Minegishi, Tatsuya Higashi, Ming-Rong Zhang

**Affiliations:** 1 Department of Advanced Nuclear Medicine Sciences, National Institutes for Quantum and Radiological Science and Technology, 4-9-1 Anagawa, Inage-Ku, Chiba, 263-8555 Japan; 2 Theranostics Research Center, Nihon Medi-Physics, Co., Ltd., Chiba, Japan; 3 Department of Molecular Imaging and Theranostics, National Institutes for Quantum and Radiological Science and Technology, Chiba, Japan

**Keywords:** Actinium-225, Radium-226, Alpha emitter, Targeted alpha therapy

## Abstract

**Purpose:**

We demonstrate cyclotron production of high-quality ^225^Ac using an electroplated ^226^Ra target.

**Methods:**

^226^Ra was extracted from legacy Ra sources using a chelating resin. Subsequent ion-exchange purification gave pure ^226^Ra with a certain amount of carrier Ba. The radium target was prepared by electroplating. We successfully deposited about 37 MBq of ^226^Ra on a target box. Maximum activation was achieved using 15.6 MeV protons on the target at 20 µA for 5 h. Two functional resins with various concentrations of nitric acid purified ^225^Ac and recovered ^226^Ra. Cooling the intermediate ^225^Ac for 2–3 weeks decayed the major byproduct of ^226^Ac and increased the radionuclidic purity of ^225^Ac. Repeating the same separation protocol provided high-quality ^225^Ac.

**Results:**

We obtained ^225^Ac at a yield of about 2.4 MBq at the end of bombardment (EOB), and the subsequent initial purification gave 1.7 MBq of ^225^Ac with ^226^Ac/^225^Ac ratio of < 3% at 4 days from EOB. Additional cooling time coupled with the separation procedure (secondary purification) effectively increased the ^225^Ac (4n + 1 series) radionuclidic purity up to 99 + %. The recovered ^225^Ac had a similar identification to commercially available ^225^Ac originating from a ^229^Th/^225^Ac generator.

**Conclusion:**

This procedure, which involves the ^226^Ra(p,2n)^225^Ac reaction and the appropriate purification, has the potential to be a major alternative pathway for ^225^Ac production because it can be performed in any facility with a compact cyclotron to address the increasing demand for ^225^Ac.

**Supplementary Information:**

The online version contains supplementary material available at 10.1007/s00259-021-05460-7.

## Introduction

**Fig. 1:**
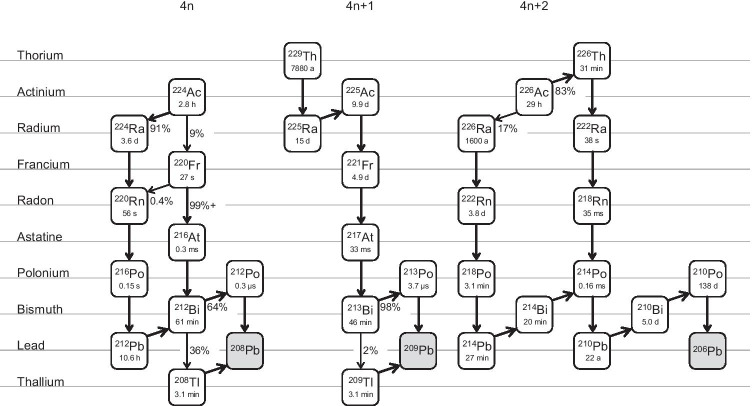
Decay chains for the 4n, 4n + 1, and 4n + 2 series

Targeted alpha therapy (TAT), which is a therapeutic regimen by radiopharmaceuticals labeled with alpha emitters, has received great interest due to its clinical impact such as ^223^RaCl_2_ and ^225^Ac-PSMA-617 [1, 2]. Compared to conventional therapy by beta emitters, alpha particles exhibit a high linear energy transfer (LET) in a short range (40–100 µm/5–9 MeV, [3]), which may provide a remarkable cytotoxic effect in a limited area of the target. Hence, unwanted radiation doses to other healthy tissues and organs may be limited. Actinium-225 (^225^Ac, α = 100%, T_1/2_ = 9.92 days) is a promising radionuclide applicable to TAT since it breeds multiple descendants via the net decays of 4α + 2β in a relatively short period (Fig. 1). This property enhances the therapeutic effects of ^225^Ac-labeled compounds [4].

The accessibility of various radioisotopes for diagnostic nuclear medicine is well established. Both accelerators and nuclear reactors are used to produce medical-grade isotopes. The former is typically used to produce short-lived isotopes, while the latter is used for large-scale, centralized production. A shortage of actinium-225 is anticipated [5] because interest has drastically increased but large-scale commercial production is still in the development phase. Currently, the most realistic path is the natural decay product by a ^229^Th/^225^Ac generator system [6, 7]. However, only a few institutes such as the Joint Research Centre (JRC, Karlsruhe, Germany), Oak Ridge National Laboratory (ORNL, TN, USA), and the Institute of Physics and Power Engineering (IPPE, Kaluga Oblast, Russia) have such capabilities and their estimated total annual capacity of actinium-225 is approximately 63 GBq (1.7 Ci) [8]. Consequently, alternative production pathways are highly desired.

Many studies have investigated increasing actinium-225 production to meet the anticipated demand. Practical options include (1) high-energy protons on ^232^Th (spallation channel, [9]), (2) moderate energy protons on ^226^Ra (nuclear transmutation channel, [10]), and (3) high-intensity gammas on ^226^Ra (photonuclear/Bremsstrahlung channel, [11–13]). Among these, option (1) holds promise because ^232^Th is not a fissile material according to nuclear regulations. However, the reaction requires a projectile with an extraordinarily high energy and intensity (e.g., 100 MeV or higher proton). Consequently, few facilities can practically produce ^225^Ac via the spallation route. On the other hand, options (2) and (3) use ^226^Ra as their target material. Option (2) may be advantageous for actinium-225 production in general facilities. However, this material is difficult to handle due to safety concerns (i.e., radon (^222^Rn) emanation and high-energy gamma emission from the descendants). For instance, as reported in [10], the reaction of ^226^Ra(p,2n)^225^Ac can be performed efficiently with a relatively small amount of ^226^Ra in a low-energy window provided by a compact medical cyclotron, *E*p ≤ 20 MeV. In this study, we evaluate the production feasibility of ^225^Ac from a ^226^Ra target that includes (1) ^226^Ra recovery from legacy needles, (2) radium target preparation, (3) activation, (4) separation, and (5) recycling of ^226^Ra. This study should be useful not only from a production capability viewpoint but also from a quality control perspective for large-scale actinium-225 production.

## Materials and methods

### Materials

Hydrochloric acid (ultra-pure, 10 M) was purchased from Kanto Chemical (Tokyo, Japan). An ammonium acetate solution (10 M) was obtained from Nacalai Tesque (Kyoto, Japan). An ammonium solution (25%), nitric acid (70%), pure water, Dowex Monosphere 550A anion exchange resin (OH form, 590 ± 50 µm), and Dowex AG1-X8 anion exchange resin (Cl form, 100–200 mesh) were obtained from FUJIFILM Wako Chemicals (Tokyo, Japan). These reagents were used as received or diluted with the appropriate volume of pure water, as needed. Chelex-100 (Na form, 100–200 mesh) was purchased from Bio-Rad Laboratories (Tokyo, Japan). It was preconditioned as the ammonium form before use. Actinium-225 nitrate (37 MBq, 99.99% radionuclidic purity) was purchased from Oak Ridge National Laboratory and used as an authentic ^225^Ac source.

### Methods

#### General

All procedures were performed in a ventilated glove box with a pressure of –50 Pa. No system for ^222^Rn handling was installed. A bag-in/out protocol with a polyethylene bag (thickness 100 µm) was employed when transferring samples across the glove box to avoid releasing ^222^Rn and other possible radioactive materials into the laboratory [Online Resource 1–3]. The maximum daily permission for handling ^226^Ra in our laboratory is 148 MBq (4 mCi).

#### Ra recovery from legacy needles

Radium needles (only information available is its size (Ø1.6 × 25 mm) and activity (1–2 mCi-^226^Ra/needle)) were sectioned into 5–6 pieces by an ordinary tube cutter (nipper type, for 1/16″ stainless tubes). The pieces, which were collected in a 50-mL glass bottle with a polypropylene screw cap (Duran Wheaton Kimble, Germany), were mixed with 3 mL of a Chelex-100 resin slurry and 7 mL of pure water. The tightly capped bottle was sonicated daily for a period of 1 week to 1 month.

Afterward, the Chelex-100 resin was filtered from the mixed materials by an empty cartridge (Bond Elut, 5 mL, Agilent Technologies, CA, USA), where the extracted ^226^Ra adsorbed on the resin. Then, 1 M HCl (5 mL) and subsequent pure water (10 mL) for rinsing were loaded into the cartridge to elute ^226^Ra from Chelex-100. The eluate was loaded into an anion exchange resin (16 mL, Monosphere) to remove chlorides. Then, the resin was washed with 10 mL pure water. The recovered solution was evaporated at 130 °C under a vacuum, yielding dried ^226^Ra in the hydroxide form.

Extending the treatment time improved the recovery efficiency of ^226^Ra. In our preliminary experiment, a maximum of 50% of the initial activity was recovered by the 1-week treatment. However, returning the mixture of needle pieces and Chelex-100 residue to the bottle again and repeating the same procedure for a month showed quantitative recovery of the initial activity. In the 1-month experiment, the slurry was adjusted to a pH ≥ 10 by adding a portion of conc. ammonium solution to ensure the Chelex-100 efficiently for absorbing ^226^Ra.

#### Target box design and target preparation

**Fig. 2:**
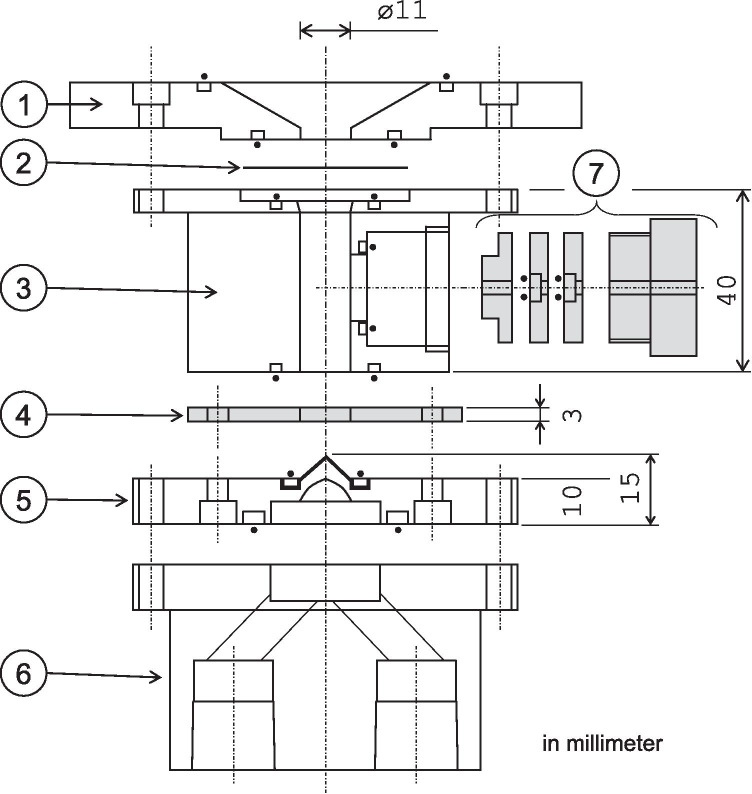
^226^Ra target assembly

The ^226^Ra target was prepared by electrodeposition. Figure 2 shows the target box assembly. A Ti cylindrical cavity (#3 in Fig. 2) with a volume of about 3.5 mL was used for the electroplating reservoir and the dissolving vessel for the Ra target after activation. A Pt rod (Ø3 mm, anode for electroplating) was held by a polyimide screw with O-rings (#7). The bottom of the Ag cavity (#5) was assembled with both #3 and a polyimide electric insulator (#4). For chemical resistance, it should be noted that the Ra-depositing surface with a conical shape (#5) was fabricated with Au by hot isostatic pressing on the Ag body.

Purified dried ^226^Ra, which ranged from 14.8 to 38.9 MBq (400–1050 µCi), was dissolved in 1 mL of 0.1 M HCl and 2 mL of 0.5 M ammonium acetate to prepare the electrolyte. The electrolyte was placed in the target box, and a constant current of 100 mA DC was applied in the pulse mode (5 Hz with a 0.1 s on–off cycle) for 3 h with a 15-mm gap between the cathode and the anode. After the electrodeposition process, the electrolyte was removed from the cavity by pipette work, and introduced in and removed from 2 mL of pure water twice to wash out the residual electrolyte in the target box. These rinsing solutions were collected as they may contain undeposited free ^226^Ra. Then, the deposition efficiency was evaluated by measuring the ^226^Ra activity. The cavity stood undisturbed overnight (> 15 h) to dry the Ra surface naturally in a ventilated glove box. Eventually, the Pt anode was withdrawn from the cavity and sealed with a thin Nb foil (50 µm). The cavity at the beam entrance was sealed with a 50-µm-thick Nb foil (#2 in Fig. 2) with #1.

#### Activation

Activations were carried out by 34 MeV H_2_^+^ (ionized molecular hydrogen) provided by NIRS-AVF-930 cyclotron at a nominal intensity of 10 µA for 3–5 h. This condition increased the intensity of the lower energy particles accelerated by a relatively larger cyclotron to give 17 MeV protons at nearly 20 µA by splitting the kinetic H_2_^+^ ion at the vacuum isolation window. The estimated proton energy on the target material by SRIM code [14] was 15.6 MeV after passing through the vacuum foil (Al, 100 µm), the He cooling layer (30 mm), and the target foil (Nb, 50 µm). To enrich the expected ^225^Ac yield, the on-target energy of 15.6 MeV was set between two energies showing the highest cross-sections for the ^226^Ra(p,2n)^225^Ac provided by the ALICE code (ca. 700 mb at 15 MeV) and the previous study (ca. 710 mb at 16.8 MeV) [10].

#### Separation of ^225^Ac from the target matrix

**Fig. 3:**
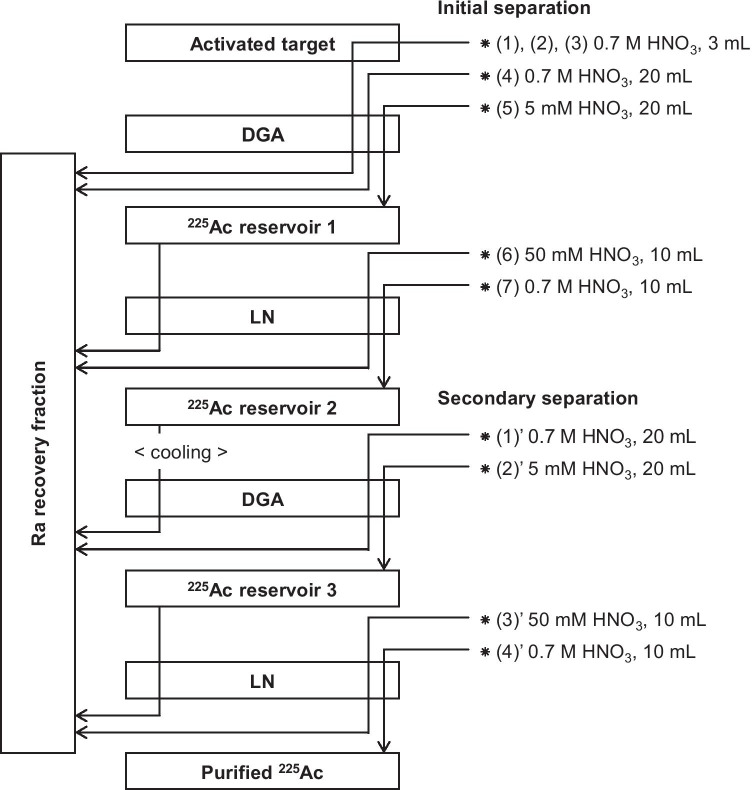
Separation diagram for ^225^Ac from the ^226^Ra matrix

Figure 3 shows our newly developed separation procedure, which was implemented 3–4 days after the end of bombardment (EOB). The activated target was dissolved in 3 mL of 0.7 M HNO_3_, and the solution was loaded slowly into a DGA cartridge (*N*,*N*,*N′*,*N′*-tetra-*n*-octyldiglycolamide, 1 mL, Eichrom Technologies, IL, USA). To increase the leftover recovery of Ac/Ra, another 3 mL of 0.7 M HNO_3_ was introduced into the target cavity and the rinsing fraction was loaded into the same DGA cartridge. This step was repeated twice.

The DGA cartridge was washed with 20 mL of 0.7 M HNO_3_ to remove residual ^226^Ra. Then, 5 mM HNO_3_ (10 mL) was loaded into the DGA to elute ^225^Ac, which was the fraction collected in an intermediate reservoir. Subsequently, the crude ^225^Ac fraction was loaded into a LN cartridge (di(2-ethylhexyl)orthophosphoric acid, 2 mL, Eichrom Technologies); the cartridge was washed with 10 mL of 50 mM HNO_3_ to eliminate trace amounts of ^226^Ra and subsequently well purged. All the above waste fractions were collected as the Ra recovery fraction, which was recycled for the next use. Eventually, ^225^Ac was stripped by loading 0.7 M HNO_3_ (10 mL) and collected into another intermediate reservoir.

The actinium-225 solution in this separation step contained ^226^Ac (β 83%, EC 17%, α 6 × 10^–3^%; T_1/2_ = 29.4 h) as a byproduct because it was unavoidably generated via the ^226^Ra(p,n)-channel in our activation condition. To increase the radionuclidic purity of ^225^Ac, the intermediate product was allowed to cool for 2–3 weeks, which is equivalent to 10 half-lives or more for ^226^Ac. After the cooling, the above separation protocol was repeated as the secondary purification.

Although the twice purified ^225^Ac was free from or had negligible ^226^Ac contamination in 0.7 M HNO_3_ (10 mL), it was too acidic for further use. Thus, an anion exchange resin (AG1-X8, 100–200 mesh, Cl form) was employed to exchange the counter anion of ^225^Ac with chloride and remove HCl from the product by evaporation of the above sample (130 °C under vacuum). The final product, which had a chemical form of ^225^AcCl_3_, was reconstituted in 100 µL of 0.1 M HCl for further use. The recovery yield of ^225^Ac was quantitative.

#### Recycling mode of Ra

All fractions possibly containing ^226^Ra were collected into a single vessel. The solution was adjusted to a pH ≥ 10 by adding a conc. ammonium solution and then loading into a column filled with the Chelex-100 resin (0.5 mL, NH_4_ form) to concentrate ^226^Ra. After washing the column with 10 mL of pure water, ^226^Ra was stripped by passing 1 M HCl (5 mL), and the eluant was led to an anion exchange resin column (16 mL, Monosphere, OH form). The Ra fraction was desalted by an anion exchanger, and an additional 10 mL of pure water was loaded to remove the residual ^226^Ra. The collected ^226^Ra with a volume of about 15 mL was subsequently evaporated at 130 °C under a vacuum to yield purified ^226^Ra in the hydroxide form, which was ready for the next use as the electrolyte.

### Sample analysis

#### Gamma spectroscopy

Samples were subjected to a HPGe detector coupled with a well-calibrated 4096-ch multi-channel analyzer (39 cm^2^, EGC15-185-R, Eurisys (Mirion Technologies), Lingolsheim, France); RZMCA, Laboratory Equipment, Ibaraki, Japan). A measurement uncertainty of 9% was obtained as a positive square root of the sum of the following contributing factors in quadrature: counting statistics (5%), geometrical error (5%), and detector efficiency (6%). Table 1 lists the decay data for the radionuclides of interest [15]. The detection limit for these nuclides was 3.7 Bq (0.1 nCi) with an acquisition period of 60,000 s or longer, which was equivalent to 1.2 × 10^–3^% of ^225^Ac activity in the most sensitive case.

**Table 1:** Production results

Run	#1	#2	#3
Beam condition (Ep = 15.6 MeV)	20 µA × 3 h	20 µA × 5 h	20 µA × 5 h
Ra deposition			
^ 226^Ra, initial electrolyte	14.5 MBq (391 µCi)	36.4 MBq (984 µCi)	38.8 MBq (1.05 mCi)
^226^Ra, deposited	13.5 MBq (366 µCi)	35.4 MBq (956 µCi)	37.5 MBq (1.01 mCi)
Deposition rate (%)	94	97	97
Nuclides of interest* in the initially purified sample (kBq, decay corrected to EOB)			
^225^Ac (150 keV, 0.6%)	522	2.23 × 10^3^	2.43 × 10^3^
^226^Ac (230 keV, 26.9%)	111	451	488
^224^Ac (215 keV, 52.3%)	Not detected	Not detected	Not detected
^226^Ra (186 keV, 3.64%)	Not detected	Not detected	Not detected
^214^Pb (352 keV, 35.6%)	Not detected	Not detected	Not detected
^214^Bi (609 keV, 45.5%)	5.2	13.5	33.3
^135^La (481 keV, 1.52%)	84.5	333	344
^140^La (487 keV, 43.9%)	0.0571	0.165	0.231
^212^Bi (727 keV, 6.58%)	Trace	Trace	Trace
^208^Tl (2615 keV, 99.8%)	Trace	Trace	Trace

^*^Nuclear data presented in parenthesis are used for quantification[15]

#### Alpha spectroscopy

An alpha spectrometer (Alpha Duo, Ametek Ortec, Oak Ridge, TN) equipped with an ion-implanted-silicon charged-particle detector (Ultra-AS 450 mm^2^, Ametek Ortec) and 4096-ch pulse-height analyzer (Maestro-32, Ametek Ortec) was used to acquire the alpha spectra. The spectrometer was calibrated with a mixed source (^148^ Ga, ^241^Am, and ^244^Cm; Eckert & Zeigler, Valencia, CA). An aliquot of the sample (about 370–1 k Bq (10–50 nCi) of ^225^Ac) was dropped on an Al sheet, and dried. The prepared sample without a cover was loaded inside the chamber. These samples were analyzed with an acquisition time of 1200 s.

## Results and discussion

### Ra liberation from legacy needles

In most cases, ^226^Ra prepared in the radium needle had a form of RaSO_4_. Although ionic compounds are typically water soluble, group II sulfates, including RaSO_4_, are practically insoluble in water. Our samples showed a nearly zero recovery of free ^226^Ra^2+^ when the Ra matrix was suspended in water or 1 M HCl, providing additional evidence that Ra was in the sulfate form. However, when Chelex-100 was allowed to sit long term, a remarkable recovery occurred. Trace amounts of Ra^2+^ were gradually liberated from the sulfate as the chelation sites tightly held Ra^2+^.

In addition, the Ra matrix remained in the small sheath cavity, even though the legacy needle was cut into small pieces. Sonication seemed to crumble the solid Ra matrix, and free Ra was effectively released from the matrix. The recovery rate of ^226^Ra was 30–50% after a week but quantitative after a month.

### Electrodeposition and activation results of ^226^Ra

The deposition yield of ^226^Ra was satisfactory. The difference of the ^226^Ra activity in the electrolyte between the initial and the post-deposition indicated that the yield ranged from 94 to 97% (Table 1). The deposited ^226^Ra layer, which contained some amount of carrier Ba, was practically insoluble upon washing after the deposition process. The washing fractions of pure water showed a very small activity of ^226^Ra (1–2% of the initial value).

**Fig. 4:**
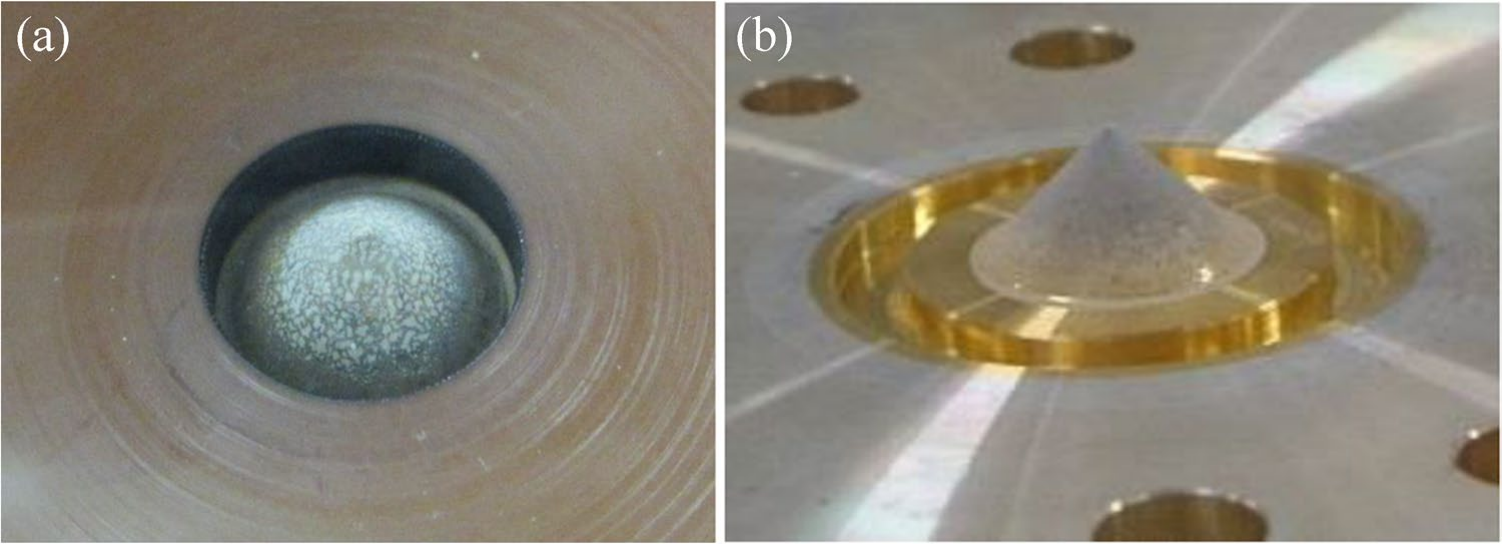
Deposition profile of (**a**) approximately 37 MBq (1 mCi) of ^226^Ra on the target surface and (**b**) 5 mg of Ba. Both electrodepositions are performed under the same conditions (100 mA constant DC for 3 h)

Figure 4 shows the electrodeposition profiles of 1-mCi of ^226^Ra (ca. 4.5 µmol) with an unknown amount of carrier and 5-mg of Ba (ca. 36 µmol) as an increasing challenge/evaluation for Ra deposition using a Ba surrogate. Due to safety regulations for ^226^Ra handling and our limited Ra inventory, this study involved a small amount of ^226^Ra. Consequently, we performed a cold experiment with Ba instead of ^226^Ra to evaluate the electrodeposition performance based on the widely accepted chemical similarity between Ra and Ba. In the case of a small amount of Ra, 13.5–37.5 MBq (366–1010 µCi) in this study, a spot-like deposition profile appeared across the cathode surface (Fig. 4a), whereas a condensed layer covered from the top of the cone to the middle of the slope in a Ba rich condition (Fig. 4b).

These results can be explained by the principle of electrodeposition. The gap between the two electro-rods around the top of the cone was the shortest and formed the best condition for electroconductivity in this system. Consequently, effective deposition was expected around the top, and most of the solute (Ra or Ba) tended to be centralized on the surface. These results are encouraging evidence to obtain rich and high-density Ra depositions when there is sufficient Ra in the electrolyte.

We obtained ^225^Ac at a yield of 522–2430 kBq (14–66 µCi, decay corrected) from a ^226^Ra target of 13.5–37.5 MBq (366–1010 µCi) irradiated by 20 µA protons for 3–5 h (Table 1). A previous study employing a ^226^Ra(p,2n)-channel reported the production yield of ^225^Ac with various amounts of ^226^Ra [10]. Although it is difficult to directly compare the previous study and our data due to the different conditions (amount of ^226^Ra prepared, chemical form, target area, target thickness, beam energy, beam intensity, and irradiation time), the previously reported yield for ^225^Ac/^226^Ra (% activity) was 13–44% without considering the activation condition. By normalizing, this reported yield of ^225^Ac was converted ca. 0.10–0.36 nCi-^225^Ac/µg-^226^Ra/(µA h) (except for the result of 12.5 µg-Ra). Our results showed 0.63–0.65 nCi-^225^Ac/µg-^226^Ra/(µA h) by applying the same correction. Considering the above-mentioned differences, both production yields are within an acceptable range, suggesting that our production capacity is similar to that of the previous study. Hence, our data support the feasibility of ^225^Ac production from an electroplated elemental ^226^Ra target. In addition, the above production index demonstrates the good stability and repeatability of our target activation capability.

### Separation

**Fig. 5:**
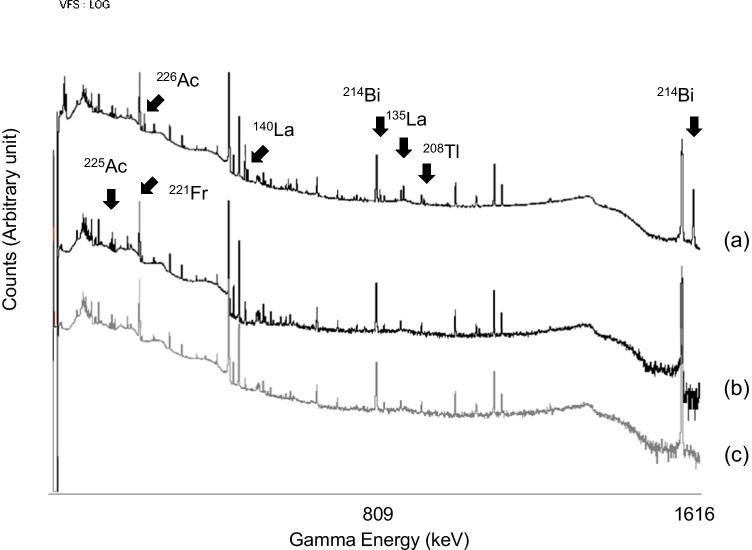
Gamma spectrum for various ^225^Ac samples: (**a**) ^225^Ac product (initial separation) after 4 days from the end of bombardment (EOB), (**b**) ^225^Ac product (secondary separation) after 20 days from EOB, and (**c**) commercially available authentic ^225^Ac (generator-made)

The initial separation of the ^225^Ac sample contained ^226^Ac and other radionuclidic impurities (Fig. 5a). Similar to ^226^Ra, ^226^Ac is a 4n + 2 series radionuclide, which generates many descendants during the cooling period (Fig. 1). Hence, repeated separation as a secondary purification removed the 4n + 2 impurities to yield high-quality ^225^Ac. Although ^224^Ac (EC 91%, α 9%, T_1/2_ = 2.8 h) should be co-produced via the ^226^Ra(p,3n)-channel (*E*_TH_ = 13.6 MeV), the half-life of ^224^Ac was too short to be detected at the end of separation at 4 days from EOB. However, the major distributions in the washing fraction and leftovers of the separation materials were a couple of ^224^Ac descendants with favorable gamma emissions in the 4n series, ^212^Bi (T_1/2_ = 61 min, 727 keV, 6.7%) and ^208^Tl (T_1/2_ = 3.1 min, 2615 keV, 99%). Moreover, trace amounts were detected in the purified actinium-225 sample, providing evidence for ^224^Ac generation. The presence of ^212^Bi and ^208^Tl in the Ac fraction was acceptable because Bi was partially similar to Ac in our separation conditions. As a result, other Bi isotopes, ^214^Bi (originating from ^226^Ra) and ^213^Bi (a descendant of ^225^Ac), should also be found in the initial actinium-225 fraction. Orphan ^214^Bi should decay upon additional cooling (Fig. 5b). On the other hand, ^212^Pb (T_1/2_ = 10.6 h, 239 keV, 44%), the parent nuclide for ^212^Bi, was not detected in the purified ^225^Ac samples. All the 4n series-nuclides with the potential to be the parent for ^212^Pb (^224^Ac–^216^Po) had shorter half-lives than ^212^Pb. The exception was ^224^Ra, which was removed along with ^226^Ra. Hence, only the 4n + 2 series was considered the byproduct in the separation process.

Other notable byproducts were ^135^La (EC, T_1/2_ = 19.5 h) and ^140^La (β, T_1/2_ = 1.68 days). The former presumably originated from a carrier of natural Ba in the legacy Ra needle via the ^135^Ba(p,n)-channel. However, the half-life of ^135^La is much shorter than that of ^225^Ac. Thus, an appropriate cooling time should gradually decrease the impact of ^135^La on the ^225^Ac even though the legacy Ra was not chemically purified. On the other hand, since the heaviest stable isotope of Ba is ^138^Ba, the atomic mass of ^140^La was too rich to be generated by proton activation, suggesting that fission on ^226^Ra may occur in our activation condition. In addition, ^140^Ba (β, T_1/2_ = 12.6 days), which is a parent nuclide for ^140^La, could also be generated as another fission product. Unfortunately, we were unable to directly confirm the presence of ^140^Ba because most of the characteristic gamma lines for ^140^Ba were close to those for ^214^Bi (RaC) and the chemical similarity between Ba and Ra. However, the initial separation of the actinium-225 fraction would practically eliminate ^140^Ba along with ^226^Ra due to chemical similarity. Indeed, orphan ^140^La in the actinium-225 fraction showed an acceptable half-life of 1.67 ± 0.10 days and decayed to a non-detectable level on the gamma spectrum by cooling for 2–3 weeks. This finding suggests that the carrier Ba in Ra needles does not affect the quality of ^225^Ac, and Ra purification from carrier Ba does not provide a practical advantage. A discussion on the counter fragments is available in [Online Resource 4].

**Fig. 6:**
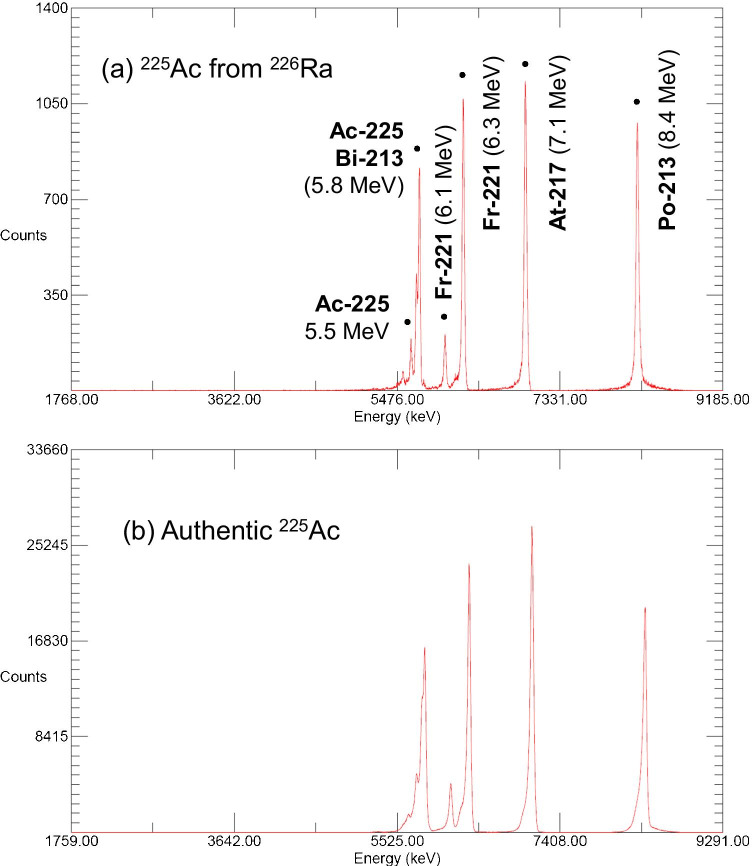
Alpha spectrum for the ^225^Ac product. Aliquot of 0.37–1.85-kBq ^225^Ac dried on an Al disk measured without a covering for (**a**) purified ^225^Ac product after 19 days from the end of bombardment (EOB) and (**b**) commercially available authentic ^225^Ac

For example, we cooled the samples for 19–20 days after EOB or 2 weeks from the end of separation. The spectra of the cooled samples were similar to that of authentic ^225^Ac originating from a ^229^Th/^225^Ac generator (Fig. 5b and c). The alpha spectrum of our ^225^Ac product also showed the same profile as the reference (Fig. 6). Notably, neither ^226^Ra (*E*α = 4.78 MeV, 94%) nor ^210^Po (*E*α = 5.30 MeV, 100%) was detected. Hence, the double separation with an appropriate cooling period gave pure ^225^Ac with a quality comparable to generator-made ^225^Ac.

### Recovery of ^226^Ra for recycling

**Fig. 7:**
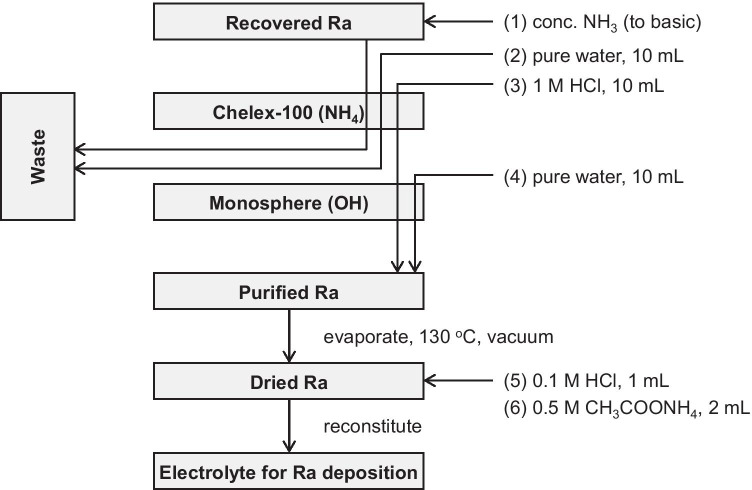
Diagram of ^226^Ra recycling

We developed a closed circuit for Ra recycling to minimize the loss of the ^226^Ra inventory (Figs. 3 and 7). This process effectively reduced long-lived radioactive wastes. After single-runs of this circuit with a 37-MBq (1-mCi) ^226^Ra, we evaluated the ^226^Ra leftover in each measurable material (i.e., the Ra recovery fraction), separation material (cartridge), and reservoir. The Ra recovery fraction contained 90–98% ^226^Ra, and other materials were negligible. For example, < 37–74 kBq (1–2 µCi) of ^226^Ra activity was found in the respective materials, depending on the volume of the residual liquid presented in the small voids. In addition, any deposition/leftover on the target box could not be estimated due to its high radioactivity. This can explain the ~ 10% discrepancy in the activity distribution. Since the target box was used repeatedly, the practical loss of ^226^Ra should be negligible.

### Radionuclidic purity—impact of ^226^Ac in ^225^Ac

Actinium-226 disintegrates by pathways of β decay (83%), EC (17%), and trace α decay (6 × 10^–3^), which breed ^226^Th (α, T_1/2_ = 30.6 min), ^226^Ra, and ^222^Fr (β, T_1/2_ = 14.2 min), respectively. Among these radioisotopes, ^226^Ra, which has the longest half-life, may be concern when preparing ^225^Ac-labeled injections. Although radiation safety related to both ^226^Ac and ^226^Ra is beyond the scope of this study, the amount of ^226^Ra generation as a decay product of ^226^Ac should be considered in discussions on clinical applications or the appropriate cooling time to address the acceptable quality on the actinium-225 product.

Actinium-226 with activities of 3.2 MBq (86 µCi) and 0.24 MBq (6.4 µCi) may generate 1.13 Bq and 0.084 Bq of ^226^Ra, respectively. These values are equivalent to ^226^Ra in the human body estimated (31 pg, where 27 pg is accumulated in the skeletal system) as well as those from dietary intake (2.3 pg/day) [16]. According to the reference [2], a maximum realistic single dose of actinium-225 injection is around 10 MBq (100 kBq/kg). Therefore, a ^226^Ac/^225^Ac sample in a 10-MBq actinium-225 injection may generate ^226^Ra equivalent to the above-mentioned reference.

While the targeted alpha therapy field has yet to establish an acceptable limit for ^227^Ac (β 98.6%, α 1.38%; T_1/2_ = 21.8 y) [17], which is a major byproduct in the spallation pathway from ^232^Th target, ^227^Ac should be a good reference for rational considerations for long-life alpha-emitting byproducts. A previous study reported that ^227^Ac was equivalent to 0.7% of ^225^Ac at the time of injection (radioactivity-based estimation) [18]. One study reported that the ^227^Ac contribution to the radiation dose delivered by an actinium-225 injection was negligible [19]. However, the presence of ^227^Ac may pose a waste disposal issue. A recent study demonstrated the production feasibility of ^225^Ac without ^227^Ac (^227^Ac/^225^Ac =  < 7.5 × 10^–5^%) using ^225^Ra (β 100%; T_1/2_ = 14.9 d), which is another spallation product from ^232^Th, as the parent nuclide [17]. This report referenced the exemption activity recommended by the International Atomic Energy Agency (IAEA) [20] for a rational discussion about the long-life radionuclide.

Although our production yields were smaller than the assumed single dose of 10-MBq ^225^Ac, the activity ratio of ^226^Ac/^225^Ac in our samples ranged 1.4–2.3% at the end of the initial separation (4 days from EOB). The ratio was comparable to the potential amount for ^226^Ra delivery via dietary intake (^226^Ac/^225^Ac = 2.4%). The ^226^Ac content of around 1–2% (100–200-kBq ^226^Ac/10-MBq ^225^Ac) was higher than the exemption activity for ^226^Ac (100 kBq, [20]) but additional cooling for several days would gradually decrease the ^226^Ac activity below the exemption. In addition, the potential ^226^Ra amount in our purified samples at any time was much lower than the exemption activity (10 kBq, [20]), suggesting that radiation risks caused by ^226^Ac would be negligible or exceedingly small, if the ^226^Ac/^225^Ac ratio is close to the range of our results.

As shown in Fig. 5b, secondary separation effectively increased the radionuclidic purity of ^225^Ac, which reached > 99% within 2–3 weeks. In the future, we plan to evaluate the biological impact of ^226^Ac since the physical decay loss of ^225^Ac during the cooling period is a critical issue in the actinium-225 industry.

## Conclusion

Actinium-225 purified from an electro-deposited ^226^Ra target with two separation columns showed an acceptable quality without byproducts. The characteristics of the purified ^225^Ac were similar to those of commercially available ^225^Ac originating from a generator system. The production results showed a linear increase in the ^225^Ac yield by increasing ^226^Ra prepared.

Consequently, increasing the ^226^Ra, beam intensity, or irradiation period can achieve the clinical requirement of ^225^Ac yield, demonstrating that the proposed production method may be a viable alternative pathway to address the increasing demand for actinium-225.

## Supplementary Information

Below is the link to the electronic supplementary material.

Supplementary file1 (DOCX 2450 KB)
