# Supplementary material for: Cyclotron production of 225Ac from an electroplated 226Ra target
Source: Eur J Nucl Med Mol Imaging. 2021 Jul 1;49(1):279–89. doi: 10.1007/s00259-021-05460-7 (PMC8712309; doi:10.1007/s00259-021-05460-7)
Supplement: Supplementary file 1 — Supplementary file1 (DOCX 2450 KB) [file 259_2021_5460_MOESM1_ESM.docx]

**Supplementary Information (Online Resource)**

**Cyclotron Production of ^225^Ac from an electroplated ^226^Ra Target**

European Journal of Nuclear Medicine and Molecular Imaging

Kotaro Nagatsu*, Hisashi Suzuki, Masami Fukada, Taku Itoh, Jun Ichinose, Yoshio Honda, Katsuyuki Minegishi, Tatsuya Higashi, Ming-Rong Zhang

Contact information for Corresponding and First Author: Kotaro Nagatsu, PhD

Dept. of Advanced Nuclear Medicine Sciences, National Institutes for Quantum and Radiological Science and Technology

4-9-1 Anagawa, Inage-Ku, Chiba 263-8555 Japan

Phone +81-43-206-4039; Facsimile +81-43-206-3261

E-mail [nagatsu.kotaro@qst.go.jp](mailto:nagatsu.kotaro@qst.go.jp)


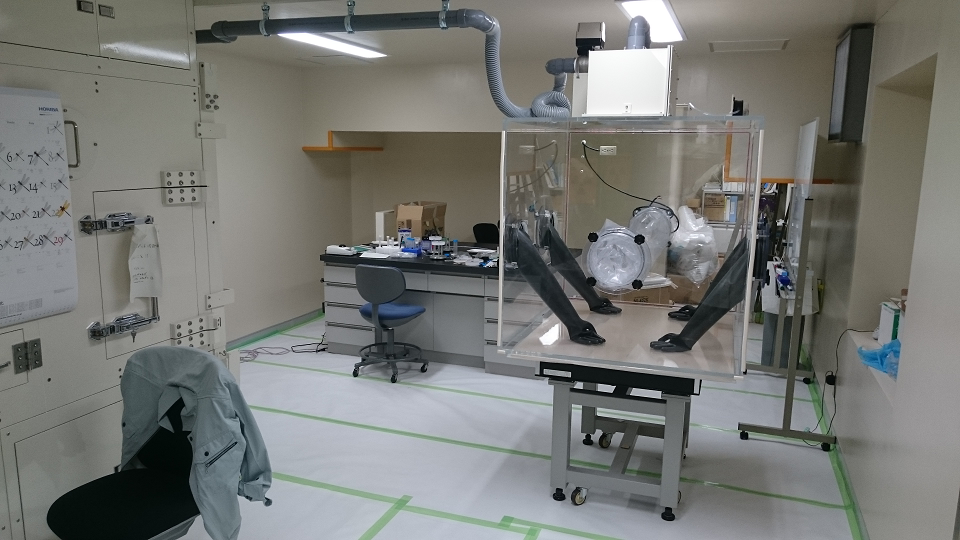


**Exhaust line**

**Always negative pressure (50 Pa)**

**No Rn-trap system**

**Access port (Φ190 mm)**

**Operate by Bag-in/-out protocol**

**Online Resource 1**

Newly installed glove box with two pairs of gloves and access ports.

All described procedures were performed inside this glove box.


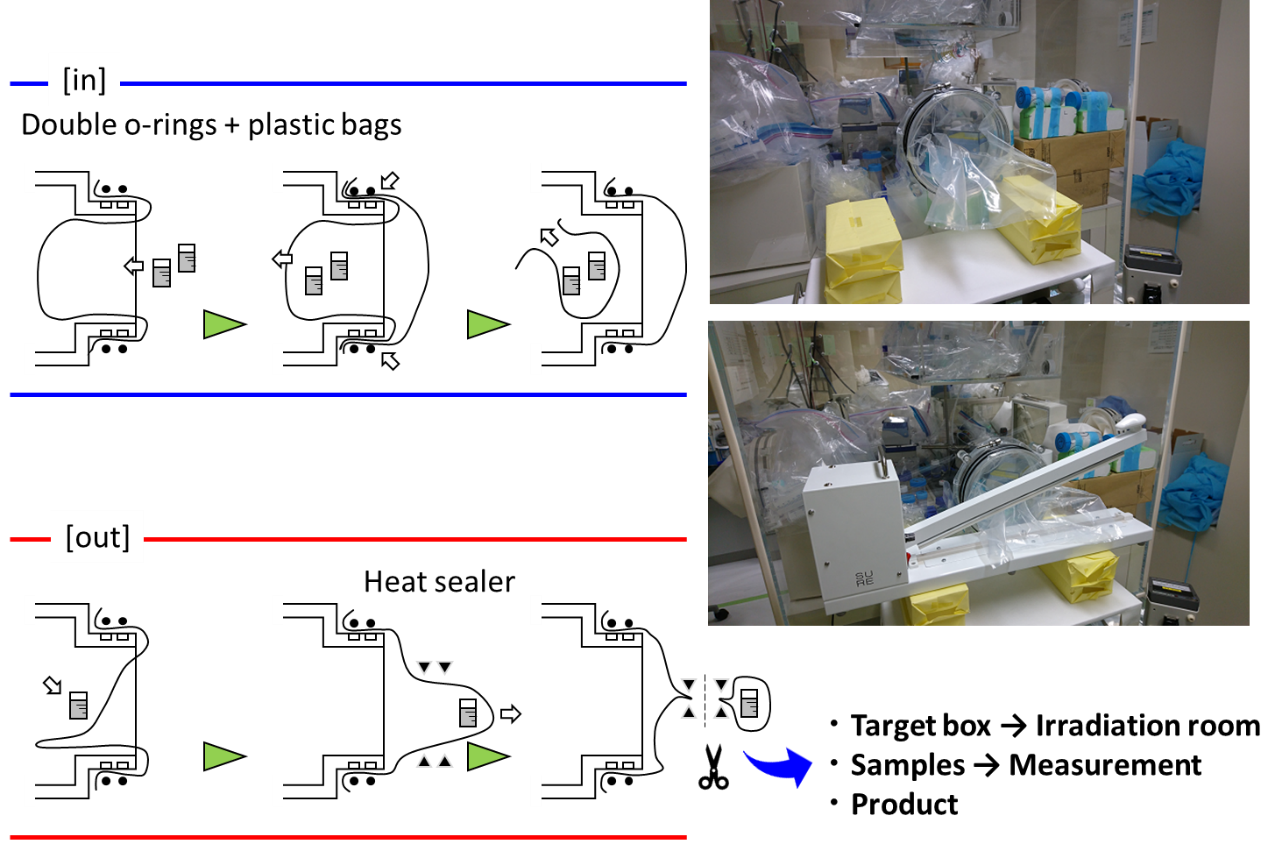


**Online Resource 2**

Bag-in/-out protocol at the sample port

All samples removed from the box were packed in polyethylene bags (100 µm) by heat sealing.


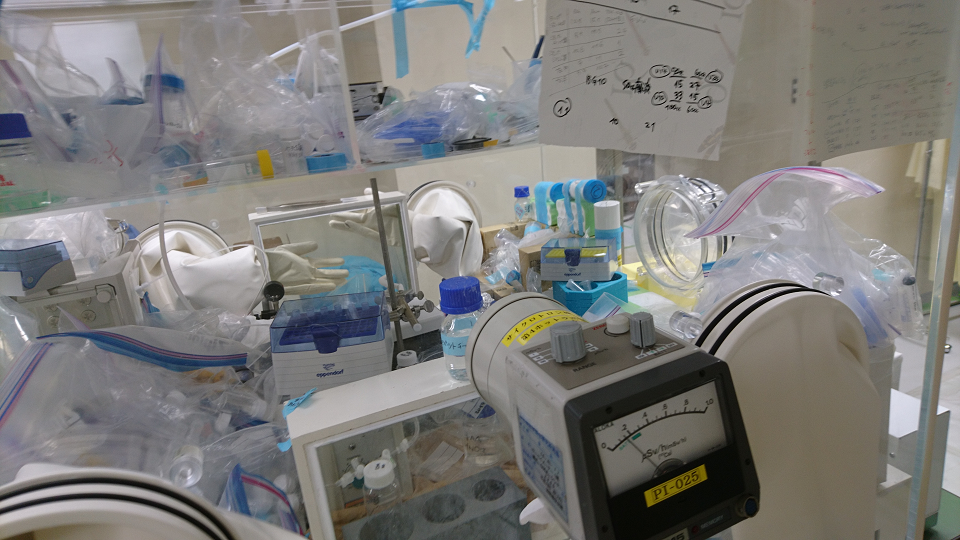


**Online Resource 3**

Typical radiation dose was about 50–70 µSv/day when 37 MBq (1 mCi)-^226^Ra was used for 6–8 h (about 50 cm away from the source through a 50-mm-lead glass shield and 10-mm-plexiglass glove box).

In this study, about 37 MBq (1 mCi) of ^226^Ra was set as the practical maximum activity to keep the receiving dose at an acceptable level. This study suggests that a remote or heavy shielded device is necessary to handle more activity of ^226^Ra.

**Online Resource 4**

Discussion on the counter fragments

Considering the proton-induced transmutation and our activation condition, ^142^Ce (11% natural abundance) may be a possible target isotope for ^140^La generation via the ^142^Ce(p,2pn)^140^La channel (threshold energy = 15.7 MeV). However, we could not find any information on Ce being added as a carrier to the preparation of Ra needles, and considering the chemical similarity, it is rational that Ba should be used for this purpose. In addition, the threshold energy for the ^142^Ce(p,2pn)^140^La and the incident energy in this study (15.6 MeV) are almost same, suggesting that the formation of ^140^La should be insufficient or negligible. As stated in the Discussion, the carrier Ba cannot contribute to the formation of ^140^La (or a potential parent nuclide, ^140^Ba). Therefore, we concluded that the ^140^La and ^140^Ba are proton-induced fission products of ^226^Ra.

As a result, the co-produced counter fragments should be _31_Ga for _57_La (and _32_Ge for _56_Ba) with atomic masses of 86 or less. However, these candidates cannot be confirmed directly due to their extremely short half-lives, which are on the order of second to milli-second. It is also rational that these counter fragments are removed simultaneously by the results of several processes for ^225^Ac purification.
